# Supplementary material for: Identification of photoperiod-induced specific miRNAs in the adrenal glands of Sunite sheep (Ovis aries)
Source: Front Vet Sci. 2022 Jul 22;9:888207. doi: 10.3389/fvets.2022.888207 (PMC9354845; doi:10.3389/fvets.2022.888207)
Supplement: Supplementary file 1 [file Data_Sheet_1.ZIP › Supplementary file/miRDeep2 file.zip!/miRDeep2 file/Novel_Structure/SPLP42A2/Novel_141.pdf]

Provisional ID	:	NC_019460.2_11228
Score total	:	13.5
Score for star read(s)	:	3.9
Score for read counts	:	6.8
Score for mfe	:	1.2
Score for randfold	:	1.6
Score for cons. seed	:	
Total read count	:	25
Mature read count	:	22
Loop read count	:	0
Star read count	:	3

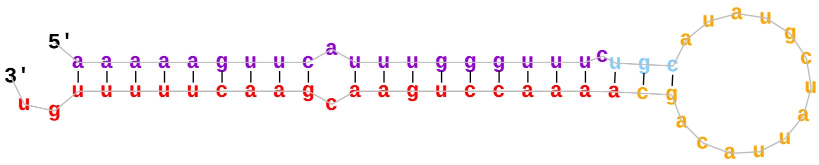

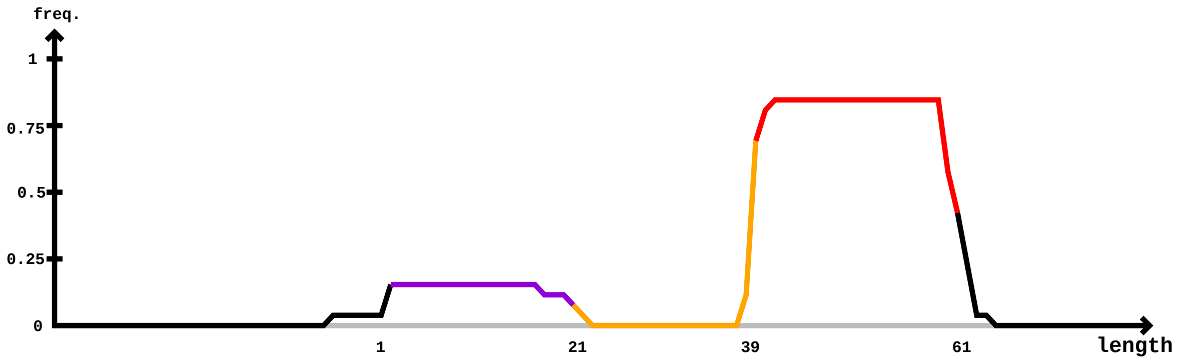

Star **Mature**

```
5'- aauuccacaagcaaugauugaauugguuuggccaaaaaguucauuuggguuucugcauaugcuauuacagcaaaaccugaacgaacuuuuuuguccaacccaauagaugcaaa   -3'    obs
    aauuccacaagcaaugauugaauugguuuggccaaaaaguucauuuggguuucugcauaugcuauuacagcaaaaccugaacgaacuuuuuuguccaacccaauagaugcaaa          exp
    .............((..(..(((((.((((.((((((((((.(((((((((.(((...............))))))))))))).)))))))))).)))).)))))..)..))..   reads      mm      sample
    ...........................uuggGcaaaaaguucauuuggg................................................................   1          1       seq
    .................................aaaaaguucauuuggguuu.............................................................   1          0       seq
    .................................aaaaaguucGuuuggguuuc............................................................   2          1       seq
    ......................................................................caaaaccugaaUgaacuuuuu......................   1          1       seq
    ......................................................................caaaaccugaacgaacuuuuuug....................   2          0       seq
    .......................................................................aaaaccugaacAaacuuuuu......................   2          1       seq
    .......................................................................aaaaccugaacUaacuuuuu......................   4          1       seq
    .......................................................................aaaaccugaacAaacuuuuuug....................   2          1       seq
    .......................................................................aaaaccugaaUgaacuuuuuugu...................   7          1       seq
    ........................................................................aaaccugaaUgaacuuuuuugu...................   3          1       seq
    .........................................................................aaccugaacgaacuuuuuugGcca................   1          1       seq
```
